# MALAT1 Fusions and Basal Cells Contribute to Primary Resistance against Androgen Receptor Inhibition in TRAMP Mice

**DOI:** 10.3390/cancers14030749

**Published:** 2022-01-31

**Authors:** Maximilian Marhold, Simon Udovica, Thais Topakian, Peter Horak, Reinhard Horvat, Erwin Tomasich, Gerwin Heller, Michael Krainer

**Affiliations:** 1 Division of Oncology, Department for Medicine I, Medical University of Vienna, A-1090 Vienna, Austria; thais.topakian@meduniwien.ac.at (T.T.); erwin.tomasich@meduniwien.ac.at (E.T.); gerwin.heller@meduniwien.ac.at (G.H.); michael.krainer@meduniwien.ac.at (M.K.); 2 Comprehensive Cancer Center Vienna, Medical University of Vienna, A-1090 Vienna, Austria; 3 Clinic of Internal Medicine I and Wilhelminen Cancer Research Institute, Klinik Ottakring, A-1090 Vienna, Austria; simon.udovica@gesundheitsverbund.at; 4 National Tumor Center (NCT), DKFZ, 69120 Heidelberg, Germany; peter.horak@nct-heidelberg.de; 5 Institute for Pathology, Medical University of Vienna, A-1090 Vienna, Austria; reinhard.horvat@meduniwien.ac.at

**Keywords:** MALAT1, prostate cancer, androgen receptor inhibition, enzalutamide, TRAMP mouse

## Abstract

**Simple Summary:**

We deeply characterized a frequently used mouse model of prostate cancer and found cellular and molecular regulators of resistance against antihormonal treatment, such as basal cell function and MALAT1 gene fusions. As these mechanisms also occur in human disease, our findings highlight the importance of this model for human cancer and may be helpful for future research focusing on overcoming antihormonal treatment resistance.

**Abstract:**

Targeting testosterone signaling through androgen deprivation therapy (ADT) or antiandrogen treatment is the standard of care for advanced prostate cancer (PCa). Although the large majority of patients initially respond to ADT and/or androgen receptor (AR) blockade, most patients suffering from advanced PCa will experience disease progression. We sought to investigate drivers of primary resistance against antiandrogen treatment in the TRAMP mouse model, an SV-40 t-antigen driven model exhibiting aggressive variants of prostate cancer, castration resistance, and neuroendocrine differentiation upon antihormonal treatment. We isolated primary tumor cell suspensions from adult male TRAMP mice and subjected them to organoid culture. Basal and non-basal cell populations were characterized by RNA sequencing, Western blotting, and quantitative real-time PCR. Furthermore, effects of androgen withdrawal and enzalutamide treatment were studied. Basal and luminal TRAMP cells exhibited distinct molecular signatures and gave rise to organoids with distinct phenotypes. TRAMP cells exhibited primary resistance against antiandrogen treatment. This was more pronounced in basal cell-derived TRAMP organoids when compared to luminal cell-derived organoids. Furthermore, we found MALAT1 gene fusions to be drivers of antiandrogen resistance in TRAMP mice through regulation of AR. Summarizing, TRAMP tumor cells exhibited primary resistance towards androgen inhibition enhanced through basal cell function and MALAT1 gene fusions.

## 1. Introduction

In developed countries, prostate cancer (PCa) is the most prevalent cancer and the second most common cause of cancer-related death in males [1]. Approximately one in seven men will be diagnosed with PCa throughout his lifetime and most patients exhibit good courses of disease with five-year survival rates of up to 99 percent [2]. Various factors contribute to whether prostate cancer patient prognosis is favorable or fatal, including comorbidities such as diabetes or hypertension [3,4,5]. However, today it is generally accepted that biology of the individual tumor and its unique molecular biological aspects—such as genetical aberrations and potentially cellular origin and heterogeneity of the tumor—play a major role in influencing patient outcome [6]. 

Tumors from patients exhibiting disease progression after or upon systemic androgen deprivation treatment (ADT), known as castration resistant prostate cancer (CRPC), often show differentiation towards an aggressive phenotype of cancer, known as neuroendocrine prostate cancer or NE-PC. For this entity, reported to be primarily resistant towards further antihormonal treatment, novel preclinical models are dearly needed for the establishment of novel treatment options as well as diagnostic and prognostic biomarkers.

Recently, using a whole exome sequencing approach, Beltran and colleagues were able to show major genetic aberrations in NE-PC [7], highlighting its unique features distinct from adenocarcinoma and CRPC [8] on a molecular level. Further, according to findings published by Aparicio et al. [9], NE-PC is caused by dual loss of the tumor suppressor genes TP53 and RB1. It was shown to be able to clonally evolve from the basal stem cell compartment of the prostate epithelium and to display a small-cell phenotype also referred to as small cell prostate cancer (SCPC) [10].

In this study, we used the Simian-Virus 40 (SV-40) T-antigen driven model of the mouse prostate (TRAMP) as a model for studying primary resistance against second generation antiandrogen treatment [11,12]. As in human disease, TRAMP carcinogenesis is caused by the dual blockade of p53 and Rb1, specifically by SV-40 t-antigens under control of a rat probasin promoter exclusively expressed in the murine prostate [13]. In this model, SCPC, metastasis, castration resistance, and neuroendocrine differentiation have been observed primarily and upon both surgical and chemical castration [14,15,16], highlighting the advantage of intratumoral heterogeneity in TRAMP mice as seen in men [17,18]. While frequency of neuroendocrine differentiation was reported to be dependent on genetic background of the mice used [19,20], it is an event commonly observed in TRAMP mice. Using this model, we aimed to identify regulators of resistance towards androgen receptor inhibition.

## 2. Materials and Methods

### 2.1. Animal Experiments

Breeding, dissection, and tumor tissue dissociation of C57BL/6-Tg(TRAMP)8247Ng/J mice (Jackson Laboratory, Bar Harbor, ME, USA) were performed as previously published by our group [21]. Cells were stained and analyzed analogously to the aforementioned published methodology. Additionally, NTRK1 (TRKA) was stained using a commercially available Alexa Fluor 647-conjugated antibody (bs-10210R-A647, Woburn, MA, USA, BIOSS antibodies). For allografting, 10,000 cells in 100 µL containing 50% Matrigel (354234, Corning, NY, USA) were injected subcutaneously into NOD.Cg-PrkdcSCIDIl2rgtm1Wjl/SzJ (NSG) mice purchased from Charles River Laboratories (Wilmington, MA, USA). 

### 2.2. Organoid Culture, Treatment, and Viability Assays

Organoid cell culture was performed analogously to the protocol by Chua et al. [22]. Following sorting into basal and luminal populations, 5000 cells were plated in each well of a 96-well ultra-low attachment plate (3474, Corning, Corning, NY, USA) using 100 µL of organoid cell culture medium. Organoid cell culture medium consisted of hepatocyte defined medium (355056, Corning, Corning, NY, USA), supplemented with 10 ng/mL epidermal growth factor ((EGF) 355056, Corning, Corning, NY, USA), 5% charcoal stripped fetal bovine serum (12676029, Thermo Fisher Scientific Inc., Waltham, MA, USA), 1× (10 µL/mL) GlutaMAX (35050, Thermo Fisher Scientific Inc., Waltham, MA, USA), 10 µM ROCK inhibitor (Y-27623, StemCell Technologies, Vancouver, BC, USA), and 5% Matrigel (354234, Corning, Corning, NY). A total of 100 nM DHT (Sigma-Aldrich, St. Louis, MO, USA) was added. A total of 10 µM enzalutamide (Lot number: RS-8BK0189-4, Astellas, Tokyo, Japan) or 10 µM abiraterone (Lot number HY-70013, Medchem, Monmouth Junction, NJ, USA) was added for drug testing experiments. A total of 0.5% DMSO (D2650, Sigma-Aldrich, St. Louis, MO, USA) was used for controls. Then, 1× Penicillin-Streptomycin (10 µL/mL, P4333, SigmaAldrich, St. Louis, MO, USA) was added. Enzalutamide was kindly provided free of charge by Astellas Pharmaceutical Inc. A total of 100 µL of freshly prepared organoid cell culture medium was added every 3 days. Organoids were grown at 37 °C, 95% humidity, and 5% CO2 for the full duration of the experiments. Organoid formation efficiency was calculated by counting visible organoids in three different wells under a Carl Zeiss Axiovert 40C light microscope (Carl Zeiss, Oberkochen, Germany). Organoid size was determined by obtaining images of at least 30 organoids in at least three different wells with a Carl Zeiss Axiovert A1 microscope (Carl Zeiss, Oberkochen, Germany) and analysis using ZEN lite software (Carl Zeiss, Oberkochen, Germany). Organoid viability was assessed using Cell Titer Glo 3D (G9681, Promega Corporation, Fitchburg, WI, USA) according to the manufacturer’s protocol. 

### 2.3. Immunohistochemistry

Organoids were washed, placed in 10% formalin and transferred into low melting agarose before paraffin embedding. Paraffin blocks were then cut into thin sections and further analyzed by a standard protocol for IHC. Following deparaffinization and rehydration with xylene and ethanol, slides were placed for 20 min at 94 °C in DEPP Epitope Retrieval Solution (DEPP-9-100, Eubio, Vienna, Austria). A blocking solution including 10% goat serum (G9023, SigmaAldrich, St. Louis, MO, USA) was applied. Slides were incubated overnight at 4 °C with primary antibodies against KRT5 (ab52635, Abcam, Cambridge, UK; 1:250), KRT8 (ab53280, Abcam, Cambridge, UK; 1:250), and AR (sc816; Santa Cruz Biotechnology, Santa Cruz, CA, USA; 1:250). After washing and endogenous peroxidase blocking using H_2_O_2_, slides were incubated with a biotinylated secondary antibody (BA-1000, Vector Laboratories, Cambridgeshire, UK; 1:200) for 30 min at RT. Staining was detected with Vectastain Elite ABC-HRP Kit (PK-6100, Vector Laboratories, Cambridgeshire, UK) and the Liquid DAB+ Substrate Chromogen System (K3468, Agilent Technologies, Santa Clara, CA, USA). Mayer’s Hemalum solution (109249, Merck, Darmstadt, Germany) was applied for counterstaining. Following dehydration with ethanol and xylene, coverslips were mounted with Eukitt mounting medium (OrsaTec, Freiburg, Germany). 

### 2.4. Western Blotting 

Organoids were collected and protein was isolated using RIPA-buffer (#sc-24948) according to the manufacturer’s protocol. Protein concentration was measured using the Pierce BCA Protein Assay Kit (#23225). Proteins were blotted on nitrocellulose membranes using a Trans-Blot TurboTM Transfer System (Bio-Rad, Hercules, CA, USA). Blots were washed with 1×TBS and blocked with 5% milk for 1 h. Membranes were incubated with primary antibodies for CK8 (Abcam, Cambridge, UK, ab53280, 1:10,000), CK5 (Abcam, Cambridge, UK, ab52635, 1:10,000), AR (Santa Cruz, sc-816, 1:200), GAPDH (Cell Signaling, Danvers, MA, USA, 5174, 1:1000), or Tubulin (Sigma, T5168, 1:1000) O/N. Membranes were washed and incubated with secondary antibodies (Cell Signaling, 7076S & 7074S, 1:1000) for 1 h. Protein was detected using Bio-Rad Clarity Western ECL (Bio-Rad, Hercules, CA, USA), according to manufacturer’s protocol.

### 2.5. RNA Sequencing and Gene Ontology Enrichment Analysis

Total RNA was isolated using RNAzol RT (R4533, Sigma-Aldrich, St. Louis, MO, USA). Quality control and cDNA synthesis was performed by the Core Facility for Genomics at the Medical University of Vienna. Raw RNA-seq data were aligned using STAR algorithm to the mouse genome version mm10. Aligned .bam files were imported into SeqMonk software (Babraham Bioinformatics, Babraham, UK) for quality control followed by differential expression analysis between groups using DeSeq2An FDR < 0.01 and a logfold change < +/−3 were defined as cutoffs for differential gene expression. Three biological replicates per group were analyzed. Gene ontology enrichment analyses were performed using the shinyGO tool [21]. Gene fusion search was performed using [22]. Data visualization was done using the R package chimeraviz 

### 2.6. qRT-PCR and PCR

RNA was extracted with RNAzol RT (R4533, Sigma-Aldrich, St. Louis, MO, USA) or Arcturus PicoPure RNA Isolation Kit (KIT0204, Thermo Fisher Scientific Inc., Waltham, MA, USA). The amount of RNA was measured on a NanoDrop 8000 spectrophotometer (Thermo Fisher Scientific Inc., Waltham, MA, USA) and transcribed with High-Capacity cDNA Reverse Transcription Kit (4368814, Thermo Fisher Scientific Inc., Waltham, MA, USA) also using RNasin Plus (N2611, Promega Corporation, Fitchburg, WI, USA). RT-PCR was performed on a Quant Studio 7 Flex Real-Time PCR System (Thermo Fisher Scientific Inc., Waltham, MA, USA) using SYBR Green PCR Master Mix (4309155, Thermo Fisher Scientific Inc., Waltham, MA, USA), mixed with UltraPure DEPC Treated Water (750023, Thermo Fisher Scientific Inc., Waltham, MA, USA) and primers. Primer sequences are available upon request. B2m was used as endogenous control. Fold changes in relative gene expression were calculated by delta Ct analysis (2-ΔΔCt).

### 2.7. TRAMPC1 Cell Culture, Lentiviral Knockdown, Treatment, and Viability Assays

Cytotoxicity of enzalutamide was measured using the CellTiter Blue assay (Promega Corporation, Fitchburg, WI, USA) by seeding 2500 cells into each well of a 96 well plate. Cells were treated at least in triplicates with 10 µM enzalutamide in 100 µL during the indicated time. After the addition of 10 µL CellTiter Blue reagent, fluorescence was measured on a Varioskan™ LUX multimode plate reader (Thermo Fisher Scientific Inc., Waltham, MA, USA).

### 2.8. MALAT1 Knockdown in TRAMPC1 Cells

Mouse MALAT1 lentiviral shRNA vectors (V3SM11247-246169059, V3SM11247-246186748, V3SM11247-246310696) and control vector (VSC11708) were purchased from Dharmacon. Packaging vectors, psPAX2 and pMD2.G, were simultaneously co-transfected with the three MALAT1 shRNA vectors or control vector into Phoenix GP cells for lentiviral production using CalPhosTM Mammalian Transfection Kit (Clontech/Takara, Kusatsu, Japan). Lentiviral supernatant was collected 48 h after transfection and concentrated with Lenti X concentrator (Takeda, Tokio, Japan) o/n according to protocol. TRAMP C1 cells were subsequently transduced and selection was performed using 2 µg/mL puromycin. Transduced TRAMPC1 cells were cultured in Dulbecco’s modified Eagle’s medium with 4 mM L-glutamine, 1.5 g/L sodium bicarbonate, 4.5 g/L glucose, 0.005 mg/mL insulin, 10 nM DHT, 10% FCS, and 5% Nu-Serum IV and passaged 1:10 every 2–3 days. shRNA sequences used can be found in Table 1:

### 2.9. Verification of MALAT1 Knockdown and Alterations in Target Gene Expression by RT-PCR

To confirm the knockdown of MALAT1 in TRAMPC1, RNA was isolated using RNAzol^®^ RT (R4533, Sigma-Aldrich) according to the manufacturer’s protocol. RNA concentration was measured on a Nanodrop 8000 Spectrophotometer (Thermo Scientific) and reversely transcribed using Lunascript RT Supermix Kit (New England Biolabs, E3010L). RT-PCR was performed on a QuantStudio 7 Flex System (Applied Biosystems) using GoTaq 2× qPCR-Mix (Promega), 500 nM primers, and 10 ng RNA/reaction. Primer sequences used were in Table 2:

### 2.10. Validation of MALAT1-Fusions by PCR

To detect NCBP3-MALAT1 fusion in TRAMPC1 cells, total RNA was isolated with RNAzol RT (Molecular Research Center IncCincinnati, OH, USA) and reversely transcribed using LunaScript RT SuperMix Kit (NEB, Ipswich, MA, USA). Primers were designed applying Primer-Blast software with the forward primer targeting NCBP3 (ACAGCGTGGAAACAACCTCA) and the reverse primer annealing to MALAT1 sequence (ACTGCTCGCTCCATCAGAAA). PCR was performed with Hot Star Taq Polymerase Kit (Qiagen, Venlo, The Netherlands). PCR products were purified using Qiaquick Gel Extraction Kit (Qiagen, Venlo, The Netherlands). Lastly, sequencing was performed. 

### 2.11. Statistical Analysis

Organoid cell culture experiments were performed as a pooled experiment of three different TRAMP tumors and repeated twice with 12 wells in each experiment, resulting in 24 replicates for both populations and treatments. Quantitative real-time PCR experiments were carried out in triplicates. Two outliers out of 72 measurements were identified and excluded from further analysis. Statistical calculations were performed using GraphPad PRISM 7 (GraphPad Software Inc, La Jolla, CA, USA). Welch’s *t*-tests were used to assess statistical significance. A two-sided *p*-value lower than 0.05 was considered as statistically significant.

## 3. Results

### 3.1. Basal and Luminal Cell-Derived Organoids from TRAMP Tumors Exhibit Heterogenous Cellular Architecture and Potency

**Figure 1:**
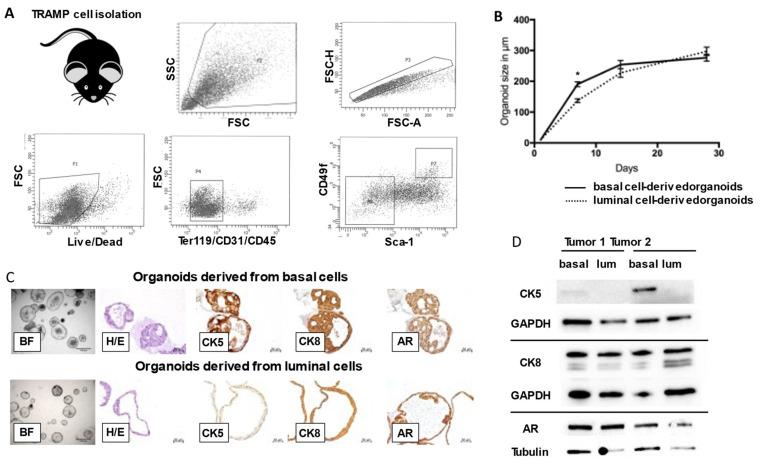
Establishment of basal cell-derived and luminal cell-derived organoid lines from TRAMP tumors. (**A**) Representative gating strategy for primary TRAMP tumor single cell suspension staining and sorting. Cells were sorted according to LIVE/DEAD yellow staining, negative blood lineage marker staining, and basal cell markers expression (Sca-1/CD49f), as previously published by our group [23]. (**B**) Basal cell-derived organoids and luminal cell-derived organoids exhibit no significant differences in maximum organoid size, although basal cell-derived organoids grow relatively faster within week one after plating. (**C**) Representative brightfield, hematoxylin/eosin, and immunohistochemistry analyses of TRAMP tumor cell-derived organoids shows differences in cellular architecture and protein expression between organoids of basal and luminal origin. Interestingly, basal cells isolated from TRAMP tumors gave rise to multilayered organoids strongly expressing the basal cell marker CK5 as well as CK8 and AR (upper panel), while luminal cell-derived organoids mainly grew as monolayered organoids not expressing CK5 (lower panel). Scale bars represent 50 µm. (**D**) Immunoblotting experiments of basal and luminal-cell derived organoid lines derived from two individual TRAMP tumors show relatively higher expression of CK5 in basal cell-derived organoids (basal) when compared to luminal cell-derived organoids (lum). No differences between organoid origins are seen in expression of the luminal cytokeratin CK8 or the androgen receptor (AR). Original Western blot can be found in File S1.

To examine differences in organoid formation between basal and luminal cell populations within TRAMP mice, we isolated basal and luminal TRAMP tumor cells using flow cytometry as previously shown by our group [23] (Figure 1A) and submitted them to organoid culture using a protocol adapted from the Shen laboratory [24] (Video S1). After sorting, we used RNA sequencing to show distinct expression patterns of cytokeratins (CK) 5 and 8 for luminal and basal cells (Figure S3B).We ensured isolation of transduced cells by performing qRT-PCR for SV40 t-antigen (Figure S3A) and injecting part of the cell suspensions obtained into NSG mice, where they grew out as allografts and formed pulmonary metastases, while retaining neuroendocrine marker expression (Figure S1D). While we observed no differences in maximum organoid size between luminal and basal cell-derived organoids (Figure 1B) we noted striking differences in cellular organoid architecture. Basal cell-derived organoids exhibited multiple layers of cells, while luminal cell-derived organoids had thinner walls that mainly consisted of one cell layer (Figure 1C). Basal cell-derived organoids exhibited expression of CK 5 and 8, with CK5 being expressed in basal cells and CK8 in luminal cells, as expected. Luminal cell-derived organoids exhibited a lack of basal cells and CK5 expression on the protein level, as confirmed by immunoblotting. Both basal and luminal cell-derived organoid lines showed androgen receptor expression (Figure 1D). 

### 3.2. Basal Cell-Derived Organoids Display Resistance towards Androgen Receptor Inhibition

**Figure 2:**
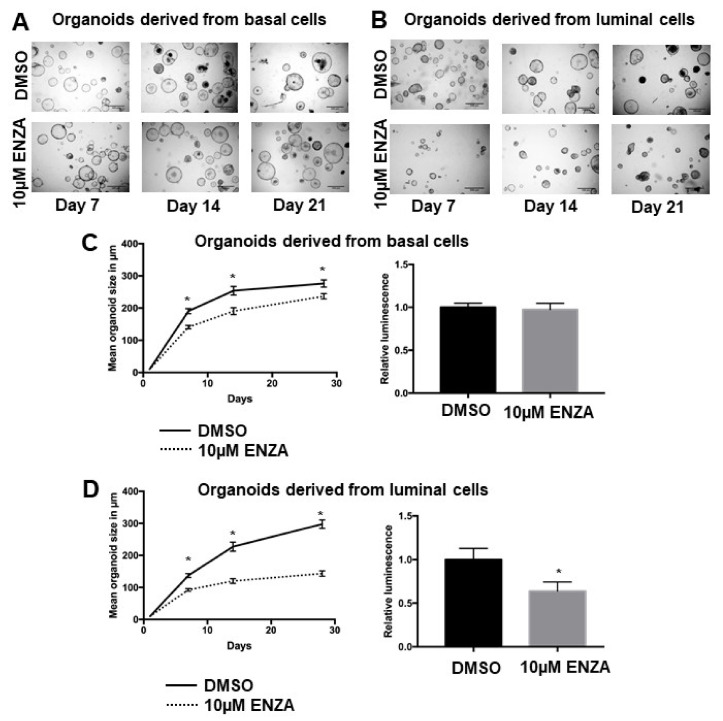
Growth patterns and viability of TRAMP tumor organoid lines. Brigthfield microscope pictures of basal cell-derived (**A**) and luminal-cell-derived (**B**) organoid cultures treated with DMSO (upper panel) or enzalutamide (lower panel). Scale bars represent 500 µm. (**C**,**D**) Both luminal and basal cell-derived organoids exhibit significantly smaller organoid size upon treatment with 10 µM enzalutamide when compared to DMSO, although decreases in organoid size are more pronounced in luminal cell-derived organoids (left panels). Interestingly, luminal cell-derived organoids but not basal cell-derived organoids exhibit lower viability as determined by luminescence upon treatment with enzalutamide. Measurements as described in the methods section of five biological replicates with three technical replicates each are shown. Error bars represent SEM ((**C**,**D**) bar graphs).

As a next step, we aimed to identify differences in responses to antiandrogen treatment of basal and luminal-cell derived organoids using the androgen receptor inhibitor enzalutamide. Interestingly, both organoid lines were able to grow under enzalutamide treatment (Figure 2). Luminal cell-derived organoids, however, exhibited significantly smaller organoid size and viability when treated with enzalutamide (Figure 2B,D). Basal cell-derived organoids on the other hand showed a small, but statistically significant decrease in size, but no decrease in viability as determined by an ATP-dependent assay (Figure 2A,C). Interestingly, no difference between luminal and basal-cell derived organoids was observed upon treatment with abiraterone (Figure S3C). 

### 3.3. Gene Expression Profiling Reveals Distinct Epithelial and Neuroendocrine Signatures for Basal and Luminal TRAMP Tumor Cells

**Figure 3:**
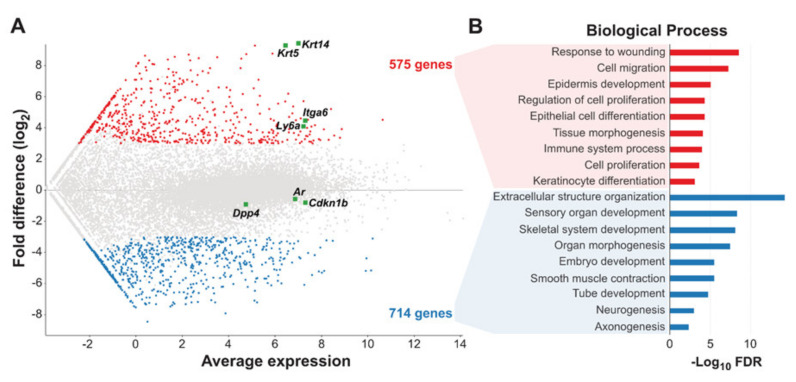
Gene expression profiling of three individual TRAMP tumors reveals distinct epithelial and neuroendocrine signatures for basal and luminal TRAMP tumor cells. (**A**) MA plot showing differentially expressed genes between basal (red) and luminal (blue) cells. Each dot represents a unique transcript. *X*-axis: average transcript expression, *Y*-axis: log2 difference in gene expression. (**B**) Barplots showing enriched biological processes in basal-specific (blue) and luminal-specific (red) genes ranked by −Log10 FDR.

Intrigued by our finding of primary resistance of basal cell-derived organoids against enzalutamide treatment, we set out to investigate gene expression signatures of basal and luminal cell populations determined by RNA sequencing of three individual TRAMP tumors. Interestingly, we found that basal and luminal cell populations exhibited distinct molecular gene expression signatures (Figure 3A). Overall, 575 and 714 basal and luminal-specific genes were identified, respectively. Gene ontology enrichment analyses of these genes showed enrichment of genes involved in response to wounding, cell migration and proliferation, epithelial differentiation, and keratinocyte differentiation in basal cells (Figure 3B), while luminal cells exhibited enrichment of genes involved mainly in developmental processes including neuronal development (Figure 3B), such as NTRK1. We confirmed the relative upregulation of NTRK1 in luminal cells compared to basal cells using qRT-PCR and the existence of NTRK1-positive cells within two individual TRAMP tumors using flow cytometry (Figure S2A). 

### 3.4. MALAT1-Fusions Are Abundant and Regulate Resistance towards Androgen Receptor Inhibition in TRAMP Tumor Cells

**Figure 4:**
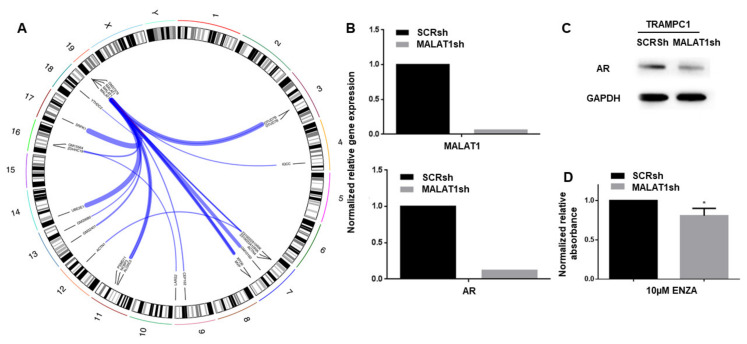
MALAT1-fusions are abundant and regulate resistance towards androgen receptor inhibition in TRAMP tumor cells. (**A**) Circos plot showing the most frequently detected gene fusions found in 12 organoid lines derived from three individual TRAMP tumors as well as the TRAMPC1 cell line (*n* = 13 samples; raw data provided in Supplementary File S1). (**B**) Lentiviral knockdown of MALAT1 (MALAT1sh) in the TRAMPC1 cell line as shown by qRT-PCR (upper bar graph) leads to downregulation of the AR (lower bar graph) in TRAMPC1 cells on the mRNA level. Values normalized to scrambled control (SCRsh). Representative experiment with three technical replicates shown. (**C**) Knockdown of MALAT1 causes downregulation of the AR in the protein level in the TRAMPC1 cell line when compared to scrambled control (SCRsh) as shown by representative western blot. Original Western blot can be found at File S1. (**D**) MALAT1 knockdown sensitizes TRAMPC1 cells to enzalutamide when compared to scrambled control, as seen through lowered relative absorbance in viability assays. Representative experiment with three technical replicates shown.

Since gene fusions—including ETS transcription factors and NTRK1—are common in human prostate cancer, we investigated whether TRAMP tumors would harbor fusion genes, again using RNA sequencing. Interestingly, we found that TRAMP tumors cells including the commercially available TRAMPC1 cell line harbored various fusion genes of MALAT1. These fusions were detectable in both luminal and basal cell-derived organoids and independent from treatment with enzalutamide (Figure 4A, Supplementary Table S1), having said that fusions were not detectable in all luminal organoid specimen upon enzalutamide treatment, partially due to low RNA yield. We confirmed the most common of these fusions, NCBP3-MALAT1, in the TRAMPC1 cell line using RT-PCR (Figure S1A). A breakpoint graph depicting this gene fusion is provided in Figure S2B. To evaluate the effects of such MALAT1 fusions on cancer cell biology in the TRAMP mouse model, we knocked down MALAT1 expression in TRAMPC1 cells using lentiviral shRNA delivery (Figure 4B). Interestingly, downregulation of MALAT1 led to downregulation of the androgen receptor on the RNA (Figure 4B, lower panel) and protein (Figure 4C) level, which correlated with higher resistance towards enzalutamide treatment (Figure 4D).

## 4. Discussion

To our knowledge, our study is the first to provide deep understanding of primary androgen receptor inhibition resistance in TRAMP mice. Primary resistance towards androgen receptor inhibition is a major problem in PCa treatment. In our study, we used the transgenic adenocarcinoma of the mouse prostate model (TRAMP mice), to identify regulators of primary resistance against androgen receptor inhibition. Advantages of this model lie in its ability to exhibit both castration resistance and primary neuroendocrine differentiation. Other murine models of PCa exhibiting neuroendocrine phenotypes and/or resistance to antiandrogen treatment [25,26,27], partially through transdifferentiation [28], exist. While these models often restrict carcinogenesis to either luminal or basal cell lineages, we found that TRAMP tumor cells of both basal and luminal phenotypes gave rise to cancer organoids in vitro (Figure 1, Figure S1D). This finding is in line with publications of both the Shen and Witte laboratories, who previously described luminal and basal cells to be potential cells of origin for prostate cancer [24,29,30]. Stoyanova et al. described that in the case of basal cells of origin, luminal cells were responsible for further tumor progression [31], which is reflected in our results, as basal cell populations vanished upon passaging in NSG mice (Figure S1B). Accordingly, we found no basal cell marker expression in subcutaneous allografts and lung metastases from TRAMP tumor cells (Figure S1D). 

Interestingly, both luminal and basal organoids exhibited primary resistance towards androgen receptor inhibition. While this effect was more pronounced in basal cell-derived cancer organoids, both organoid lines were able to form and grow out under the presence of the androgen receptor inhibitor enzalutamide (Figure 2). The same was true for treatment with abiraterone, having said that, for these treatments, no differences in viability between basal cell- and luminal cell-derived organoids were observed (Figure S3C). It is known that both basal and luminal cells respond to androgen withdrawal and stimulation and express the androgen receptor [32]. To find factors that contributed to this resistance, we performed RNA sequencing (RNAseq) experiments and provide gene expression data for both luminal and basal TRAMP tumor cells (Figure 3A). Interestingly, ETS family members such as ERG, ETV1, or ETV4 were upregulated in basal cells as compared to luminal cells (Figure S1C). 

A potential weakness of our study is the cell isolation protocol used, which did not allow sorting of different luminal cell populations. This is of some concern, as recently, Karthaus et al. [33] as well as Crowley et al. [34] reported heterogeneity within luminal, but not basal cells of the murine prostate using single-cell RNA sequencing approaches. More research is needed to understand the function of these subsets within the luminal cell compartment of murine prostate cancer models such as TRAMP mice. 

Another potential weakness of our methodology may be caused by selection of epithelial (wild-type) cells through organoid culture per se. We tried to minimize this error by ensuring that cell solutions used for organoid cultures contained tumor-initiating cells (Figure S1) and that organoids expressed the SV40 t-antigen (Figure S3A). Still, we cannot completely rule out contamination of our TRAMP tumor organoid lines with benign epithelial cells.

Furthermore, organoid experiments in our laboratory were conducted with EGF-containing cell culture medium and TRAMP cells known to express wild-type PTEN only. As a previously described crosstalk between AR and EGF [35] will potentially influence the response to AR inhibition, we argue that future experiments with EGF-deprived cell culture medium and/or organoids gained from mouse models exhibiting loss of PTEN are needed.

As fusion genes were shown to be of paramount importance in human prostate cancer and androgen signaling [36], we searched for fusion genes using RNAseq. We found fusions of MALAT1 with various genes in organoid lines of both luminal and basal origin as well as in the TRAMPC1 cell line and confirmed their existence using RT-PCR (Figure 4). MALAT1, a long non-coding RNA often expressed and thought to deregulate RNA splicing in CRPC patients [37], was shown to be a regulator of androgen receptor expression, to mediate cancer cell growth, invasion and migration and to correlate with PSA values and Gleason grading [38,39,40] in prostate cancer. Of note, a similar downregulation of the AR upon knockdown of MALAT1 was shown by Dai and colleagues in LnCAP cells [40]. Furthermore, studies found MALAT1 to be a potential diagnostic or prognostic biomarker in PCa [41,42,43,44] and a potential therapeutic target in various cancer entities [45,46]. Recently, in prostate cancer, MALAT1 was proposed to be a mediator of enzalutamide resistance through its indispensable role in AR-splice variant 7 (AR-V7) formation [47], and hence a potential target for pharmacological intervention in prostate cancer [48]. According to preclinical research by Chou et al., Cis- and Carboplatin-mediated suppression of the MALAT1/SF2 RNA splicing complex may lead to degradation of AR-V7, potentially re-sensitizing AR-V7 expressing PCa cells to enzalutamide [49]. Docetaxel treatment, on the other hand, may increase the generation of AR-V7 via altering the MALAT1/SF2 complex, potentially causing enzalutamide resistance [50]. Further, targeting MALAT1 was shown to change PCa cell metabolism towards a more glycolytic phenotype and to decrease the expression of oxidative phosphorylation enzymes causing cell arrest and death by Nanni et al. [51]. Among others, underlying mechanisms for these effects of MALAT1 in PCa were shown to be upregulation of miR-140 [52], deregulation of miR-1 and KRAS [53], enhancing function of EZH2 [54], as well as association with estrogen receptor subunits on the chromatin level [55]. The aforementioned research efforts dealing with MALAT1 and its role in regulating AR-V7, all mainly carried out in vitro, shows a need for PCa animal models expressing MALAT1.

Of note, two of the genes we found to be forming fusion genes with MALAT1, namely MVP and NCBP3, were shown to play a role in nucleocytoplasmic transport [56]. MVP, the major vault protein, was shown to be linked to multidrug resistance in a series of cancers [57] including PCa [58], and was recently proposed as a biomarker for lethal outcomes in PCa by Ramberg and colleagues [59]. To our current knowledge, it is unknown whether MALAT1 fusions occur in men, and whether they would play a role in resistance to enzalutamide or development of neuroendocrine phenotypes such as NEPC in PCa. We therefore highlight the importance of future studies evaluating the clinical impact of MALAT1 overexpression and/or gene fusions in human PCa.

## 5. Conclusions

We highlight the role of TRAMP mice as a model of studying MALAT1-driven prostate cancer and primary second-generation antiandrogen resistance. 

## Figures and Tables

**Table 1:** shRNA sequences.

V3SM11247-246169059	AAAAGGCTCGTTCACCTGT
V3SM11247-246186748	TGCGATTTCCTCGGGCTGA
V3SM11247-246310696	AACCCTACTGACGAATCTG

**Table 2:** Primer sequences.

	Fwd	Rev
MALAT1	TGCTGCATTAAGCCTGGAGT	ACGAAACATTGGCACACTGC
AR	AATGAGTACCGCATGCACAA	CCCATCCACTGGAATAATGC
ACTIN	ATGAGCTGCCTGACGGCCAGGTCATC	TGGTACCACCAGACAGCACTGTGTTG

## Data Availability

The data presented in this study are available on request from the corresponding author. The data are not publicly available due to privacy issues.

## Supplementary Materials

The following are available online at www.mdpi.com/article/10.3390/cancers14030749/s1, Figure S1: (A) Verification of the NCBP3-MALAT1 fusion in TRAMPC1 cells using PCR. The PCR product yields a band of predicted length. DNA ladder and H20 control lane shown. (B) Two rounds of passaging of TRAMP tumor cells as allografts in NSG mice confirm tumor-inducing properties of TRAMP cells. The CD49fhi/Sca-1hi population is lost through propagation in vivo. (C) Differential expression of genes of interest between basal (red lanes, left) and luminal (blue lanes, red) cells isolated from three individual TRAMP tumors as evaluated using RNA-sequencing. (D) TRAMP allografts in NSG mice. Slide overview of subcutaneous allograft and host animal lung (outer left and middle left picture). H/E and immunohistochemistry (CK8, neuroendocrine markers SynA/CgA, CK5) microscope pictures of subcutaneous graft (upper panel) and lung metastasis (lower panel) of TRAMP allografts in NSG mice, Figure S2: (A) Flow cytometry analyses of cell suspensions from two individual TRAMP tumors show cell populations expressing the neuroendocrine marker NTRK1. (B) Fusion gene breakpoint graph of the NCBP3-MALAT1 fusion as found in the TRAMPC1 cell line using DNA sequencing, Figure S3: (A) Presence of the SV-40 t-antigen in luminal cell- and basal cell-derived organoids shown after loading RT-PCR products in agarose gel and following electrophoresis. Expected product length for SV-40 t-antigen was 70 bp. DNA ladder shown. B2M was used as a control. (B) Absolute expression values (reads) from RNAseq experiments of freshly isolated and FACS-sorted basal and luminal TRAMP tumor cells as described in Figure 1A. Note abundant expression of CK5 in basal cells and lack of expression of CK5 in luminal cells. (C) Treatment of basal cell- and luminal cell-derived tumor organoids with 10 µM abiraterone for 72 h. Table S1: List of gene fusions found, Video S1: Video showing growth of TRAMP organoids, File S1: Original western blots.
