# Supplementary figures and images for: MALAT1 Fusions and Basal Cells Contribute to Primary Resistance against Androgen Receptor Inhibition in TRAMP Mice
Source: Cancers (Basel). 2022 Jan 31;14(3):749. doi: 10.3390/cancers14030749 (PMC8833778; doi:10.3390/cancers14030749)

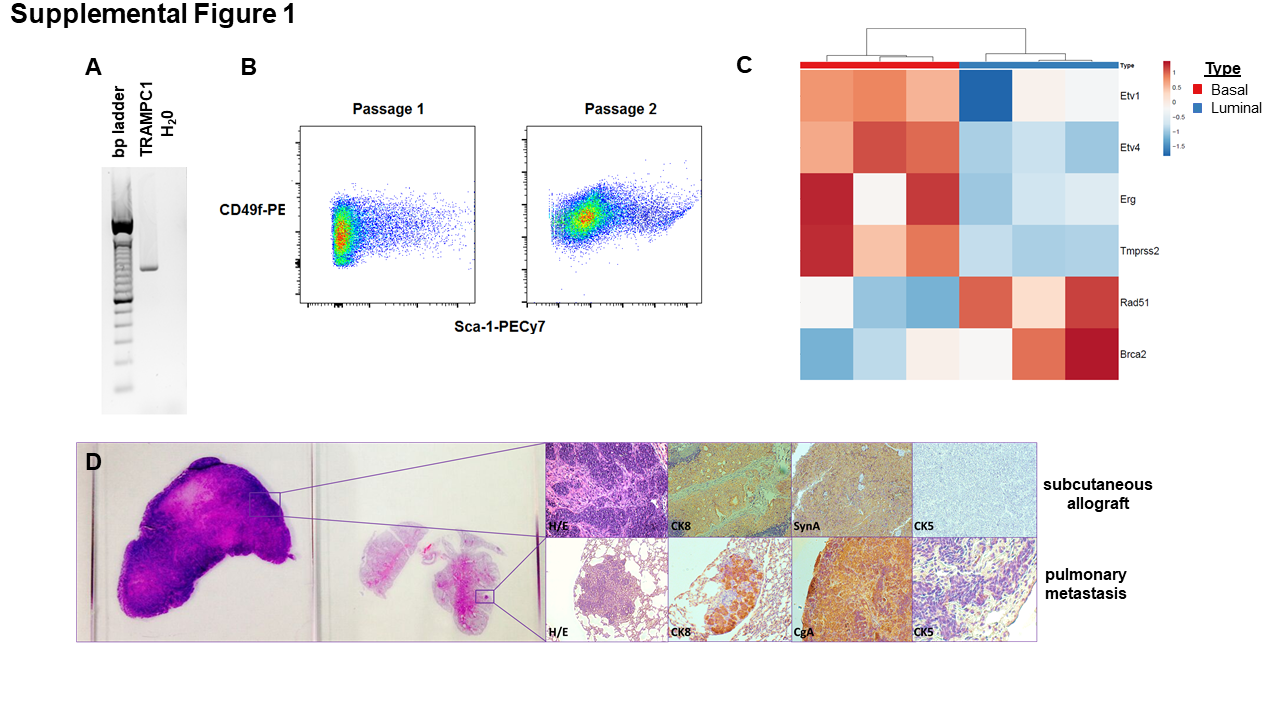

Supplement: Supplementary file 1 [file cancers-14-00749-s001.zip › FigureS1.TIF]

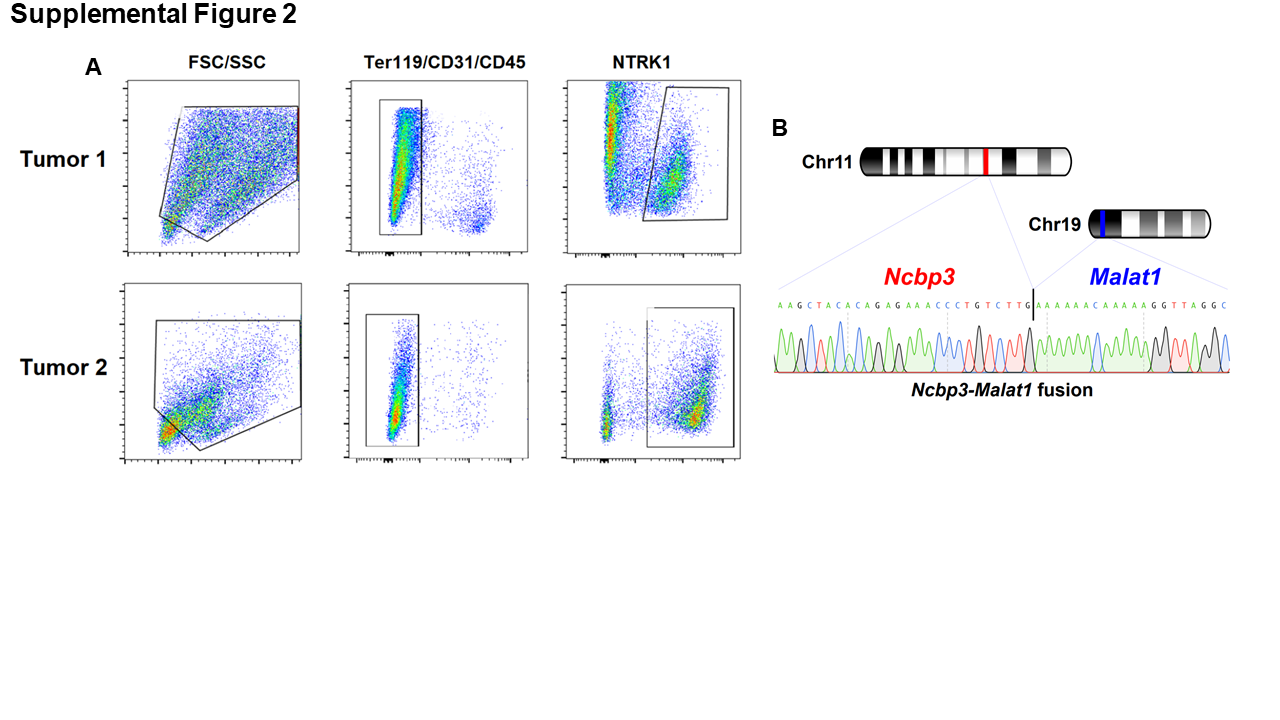

Supplement: Supplementary file 1 [file cancers-14-00749-s001.zip › FigureS2.tif]

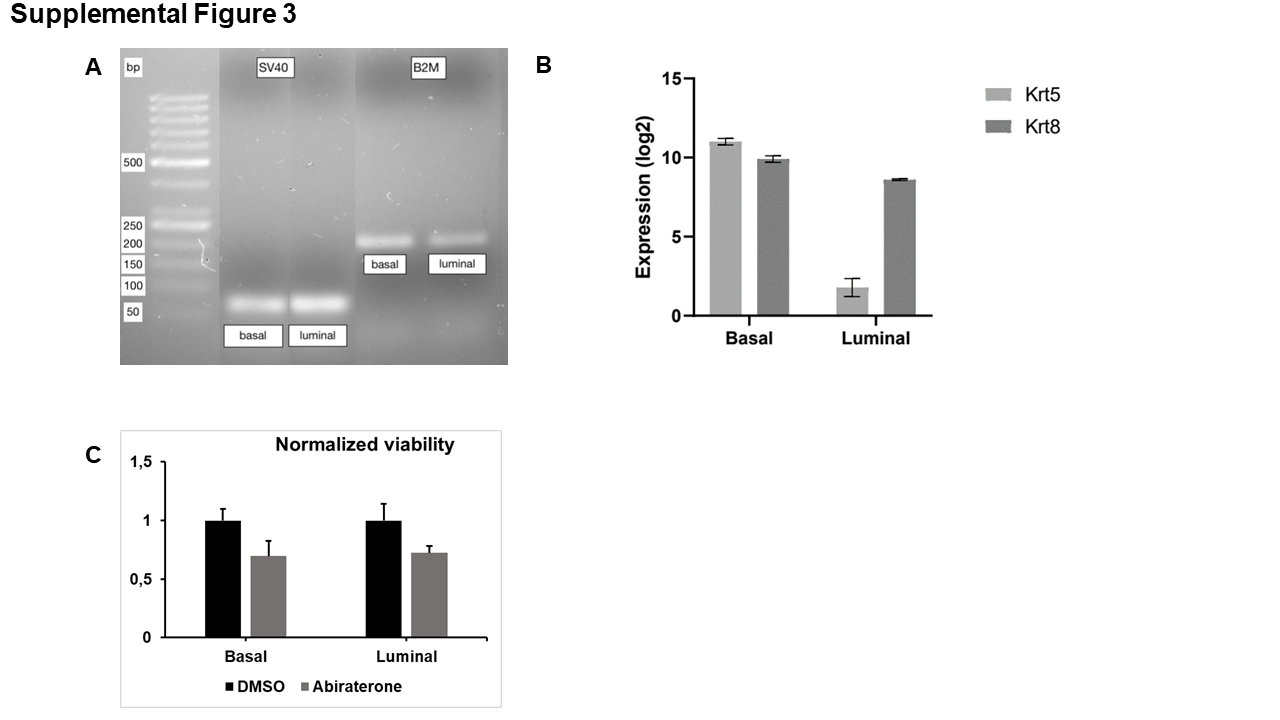

Supplement: Supplementary file 1 [file cancers-14-00749-s001.zip › FigureS3.tif]

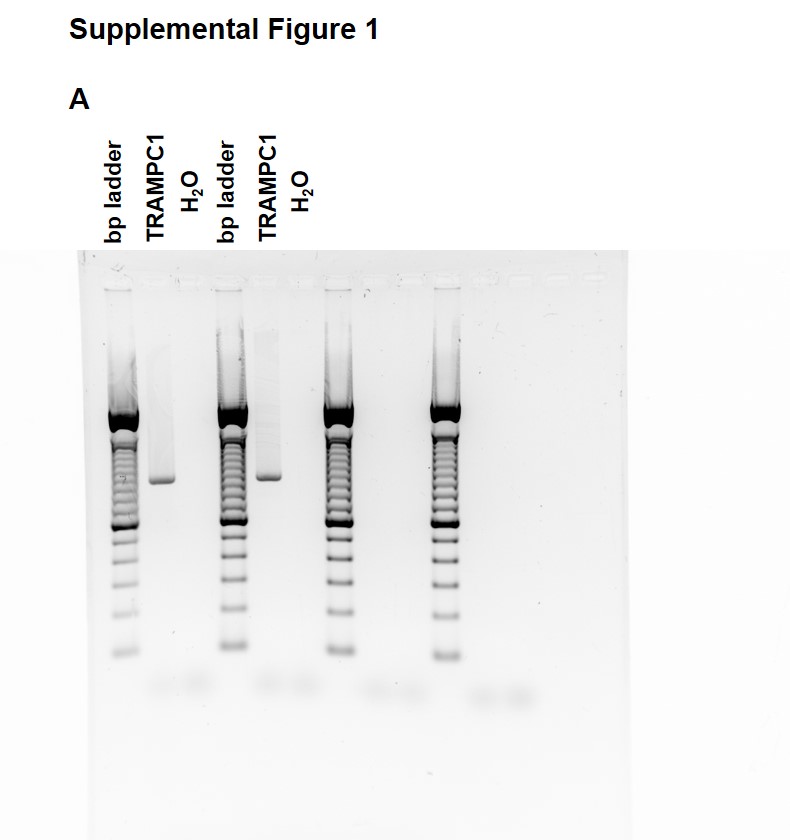

Supplement: Supplementary file 1 [file cancers-14-00749-s001.zip › File S1/For Fig1A.jpg]

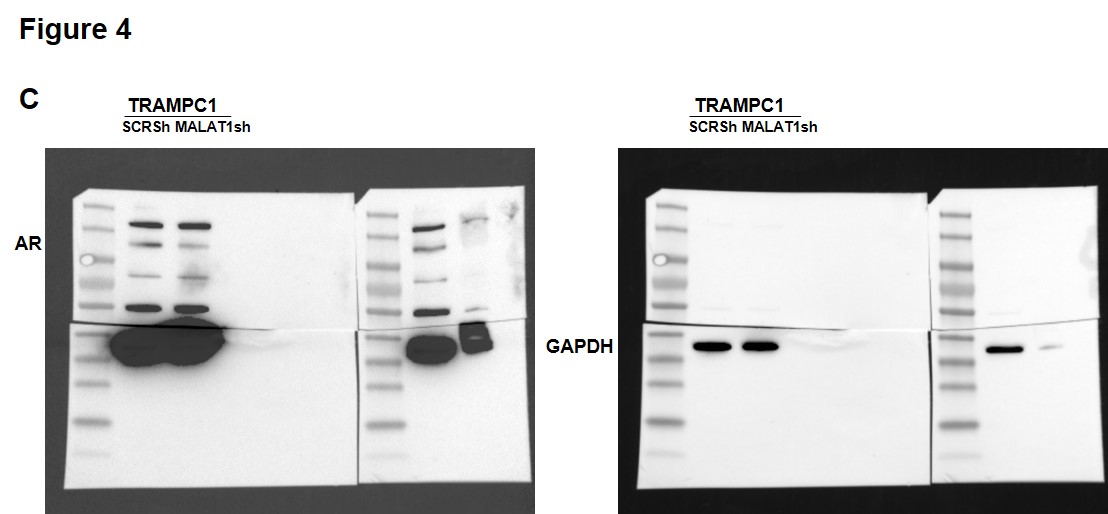

Supplement: Supplementary file 1 [file cancers-14-00749-s001.zip › File S1/For Fig4C.jpg]

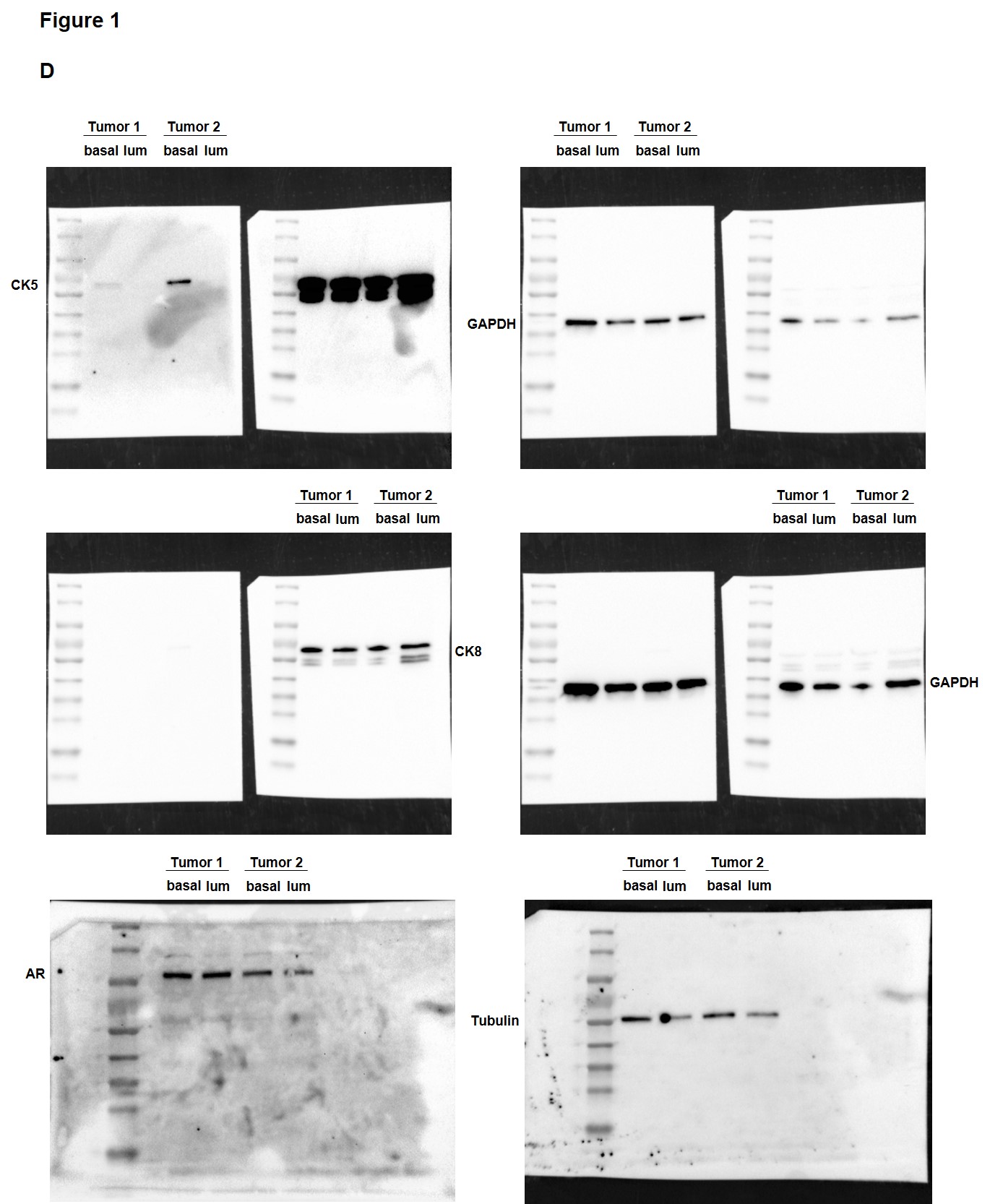

Supplement: Supplementary file 1 [file cancers-14-00749-s001.zip › File S1/For Figure1D.jpg]
